# A Method for Underwater Wireless Data Transmission in a Hydroacoustic Channel under NLOS Conditions

**DOI:** 10.3390/s21237825

**Published:** 2021-11-24

**Authors:** Jerzy Mizeraczyk, Ryszard Studanski, Andrzej Zak, Agnieszka Czapiewska

**Affiliations:** 1 Faculty of Electrical Engineering, Gdynia Maritime University, Morska 81-87, 81-255 Gdynia, Poland; j.mizeraczyk@we.umg.edu.pl (J.M.); r.studanski@we.umg.edu.pl (R.S.); 2 Faculty of Mechanical and Electrical Engineering, Polish Naval Academy, Smidowicz 69, 81-127 Gdynia, Poland; a.zak@amw.gdynia.pl; 3 Faculty of Electronics, Telecommunications and Informatics, Gdansk University of Technology, Narutowicza 11/12, 80-233 Gdansk, Poland

**Keywords:** underwater communication, NLOS, MFSK, data transmission, hydroacoustic channel, diversity combining

## Abstract

Wireless data transmission in the hydroacoustic channel under non-line-of-sight (NLOS) propagation conditions, for example, during a wreck penetration, is difficult to implement reliably. This is mostly due to the multipath propagation, which causes a reduction in the quality of data reception. Therefore, in this work an attempt has been made to develop a reliable method of wireless underwater communication test it under the NLOS conditions. In our method, we used multiple frequency-shift keying (MFSK) modulation, sending a single bit on two carriers, and diversity combining. The method was tested in laboratory conditions which simulated underwater signal propagation during the penetration of the wreck. The propagation conditions were investigated by determining the impulse responses at selected measurement points using the correlation method. Additionally, for comparison, the data transmission quality was determined by the bit error rate (BER) under the same conditions using direct sequence spread spectrum (DSSS) and binary phase shift keying (BPSK) modulation. The obtained results confirmed the usefulness of the application of the developed method for wireless data transmission in a hydroacoustic channel under NLOS conditions.

## 1. Introduction

The development of underwater robotics and environmental monitoring systems has created the need to provide wireless communication in the water. In addition, there are the needs of the military and state services, e.g., in the field of minefield control or surveillance systems in strategic areas, such as ports [1]. Despite significant progress in the development of telecommunications systems, wireless communication in the aquatic environment still causes many problems. In such an environment, acoustic waves propagate the best, so it is primarily used as a transmission medium. Unfortunately, the propagation of a sound wave in water is accompanied by many unfavorable phenomena that affect the overall quality of transmission expressed by the range of data transmission, bit error rate or bit rate. Among these phenomena, the most important are the multipath propagation leading to intersymbol interference and the Doppler effect which, due to the relatively low speed of sound propagation in the water environment, is hundreds of thousands of times stronger than that it is in the case of radio communication [2]. Besides, serious difficulties exist in obtaining relatively fast (about kilobits) and reliable (BER about 10^−3^) transmission are caused by waters with intensive hydrotechnical infrastructure, such as ports [3]. Communication under NLOS conditions is a special case. Ensuring such communication is necessary, for example, when exploring wrecks. Often, such exploration is performed by a diver or with the use of an underwater vehicle does not allow the use of cables due to the need to enter into the wreckage and the high risk of entanglement or deadlock of the transmitting cables. In this case, the complex layout of the corridors in the wreckage requires wireless communication. Such communication must be ensured with a relatively low bit error rate of 10^−2^–10^−3^ and a relatively high bit rate, in the range of kilobits, which would allow, for example, with the use of appropriate vocoders, for voice communication with the diver. Unfortunately, currently proposed commercial solutions do not allow communication under NLOS conditions with such parameters. Therefore, this work is aimed at developing a method of wireless underwater communication under NLOS conditions.

Generally, there are three methods of wireless transmission in water, described in the literature. Radio frequency (RF) transmission is possible at very short distances in sensor networks [4]. Optical communication, of great interest in recent years [5,6], is also feasible as a technique that promises high throughputs over long distances. However, at present most research is still carried out under line-of-sight (LOS) laboratory conditions. The authors of this publication found one article discussing wireless optical communication in water under NLOS conditions [7]. However, the tests in [7] were carried out only in laboratory conditions, in which the distance between the receiver and the transmitter was only 90 cm. The third method is acoustic underwater communication, which has a long history [8]. The physical phenomena associated with it are well known, what allows for the creation of simulators of hydroacoustic channels based on measurements carried out in various reservoirs and with various measurement methods [9]. Despite the extensive knowledge about the phenomena occurring during the propagation of acoustic waves in water [2,10,11,12], this environment is still a challenge in the field of designing underwater wireless communication systems. This is mainly due to the high variability of the propagation environment over time, especially in shallow waters [13], hence the great interest of reliable and efficient methods of wireless acoustic communication underwater. In the literature, the wireless underwater acoustic communication systems using linear frequency modulation [14], BPSK, QPSK and 4-QAM modulations [15,16,17], as well as proposals for the use of the OFDM technique [18,19] can be found. However, the authors of those system worked under LOS conditions and did not checked the performance of their systems under NLOS conditions.

The further structure of this paper is as follows: the second chapter presents the method of data transmission developed by us, the third chapter describes the channel model, chapter four describes the test stand and presents the results of tests carried out in simulations and laboratory conditions, and chapter five contains conclusions from the research and proposes further research towards the development of the underwater acoustic communication system.

## 2. Proposed Method of Data Transmission

The data transmission set used in this work consists of two parts, transmitting and receiving. It operates in the single input multiple output (SIMO) mode, i.e., the transmitter uses a single transmitting hydrophone as a projector, and the receiver has a set of three receiving hydrophones in fixed spatial configuration. The basic parameters of the data transmission system are as follows:

$N_{sf}$—the number of symbol in a frame.
$N_{bs}$—the number of bits in one symbol.
$F_{d}$—the lower frequency of the bandwidth (in Hz).
$\Delta f_{t}$—the frequency resolution of the spectrum in the transmitter (in Hz).
$\Delta f_{bits}$—the frequency interval between bits (in Hz).
$\Delta f_{r}$—the desired frequency resolution at the receiver (in Hz).
$\tau_{guard}$—the guard interval (in sec).
$F_{c_{preamble}}$—the preamble carrier frequency.
$N_{preamble}$—the preamble sequence length.
$S_{preamble}$—the preamble modulation rate.

These parameters defining the communication conditions, are set prior to transmission. They are available to both the transmitter and the receiver. In addition, their change allows the system to be adapted to the need of the use and the conditions of the sound wave propagation in water.

**Figure 1:**
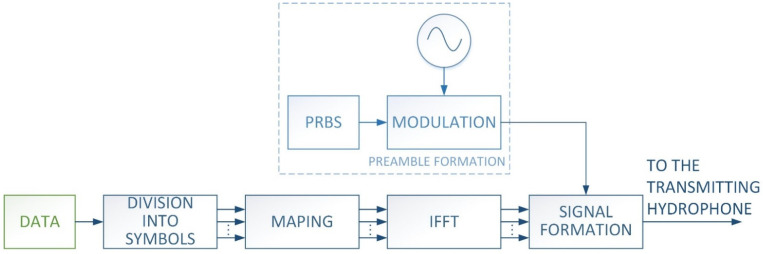
Block diagram of the transmitter.

The transmitter works in accordance with the block diagram shown in Figure 1.

**Figure 2:**
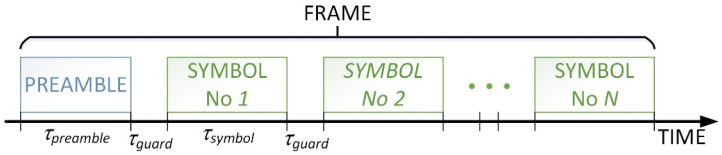
Signal form $\tau_{premable}$—duration of the preamble, $\tau_{guard}$ —guard interval, $\tau_{symbol}$ —duration of the symbol.

The binary data is divided into individual symbols depending on the number of symbols in the frame and the number of bits transmitted in one symbol. After being divided into symbols, each symbol is mapped to the frequencies. Before the mapping begins, a frequency grid is generated based on the configuration data, i.e., the lower frequency of the bandwidth, the frequency resolution of the spectrum at the transmitter, the number of bits transmitted in the symbol, and the frequency interval between bits. In this work, the MFSK modulation in which each bit is mapped to two frequencies was used. In the used MFSK modulation technique a detection threshold at the receiver is not required, moreover the transmitted signal power does not depend on the symbol form. For a pair of frequencies assigned to a bit, the signal is emitted on one of them, depending on the value of the bit. Forcing the emission is carried out by entering the value 1 or 0 on the appropriate spectrum component occupied by a given bit. This produces a vector that maps data to frequencies. In the next step, an inverse fast Fourier transform (IFFT) is performed on the vectors previously formed for each symbol, with a random phase assumed for each spectral component. The phase randomization aims to evenly distribute the instantaneous values of signal in the time domain and simultaneous reducing the probability of periodic signal amplification. The next step is forming the output signal, which consists in the appropriate time setting of the individual symbols in relation to each other, as well as adding the so-called preamble. The preamble is generated based on a known pseudo-random binary sequence (PRBS) of a predetermined length that modulates the phase of a sinusoidal signal with the predetermined carrier frequency. The final form of the signal is shown in Figure 2.

After the preamble, and between the individual symbols, a defined waiting time $\tau_{guard}$ is used to enable the just-transmitted signal to fade out in the propagation environment. This is to prevent any interference between the individual symbols. The presence of the previous signal in the propagation environment during the transmission of the next one may lead to inter-symbol interference and ultimately errors in reception. In the presented case it was assumed that the guard interval is longer than the channel memory time.

**Figure 3:**
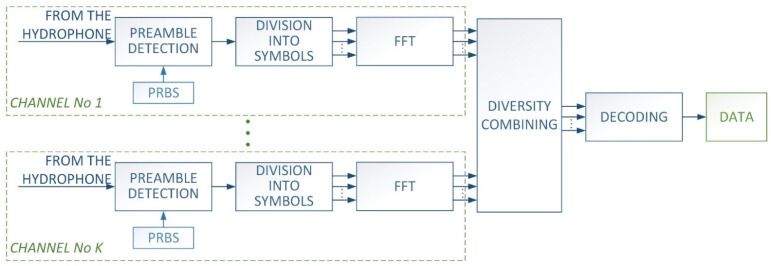
Block diagram of receiver.

In the receiver, the process is opposite of that in the transmitter. Figure 3 shows the block diagram of the receiver. After recording the signal, an attempt is made to detect the preamble.

It consists in correlating the signal transferred to the baseband with the known PRBS, what can be written in the form [3]:

(1) $$\left|h\left(k\right)\right|=\sqrt{{\left(\overset{N-1-\left|k\right|}{\underset{n=0}{\sum }}z\left(n\right)y_{s}\left(n-k\right)\right)}^{2}+{\left(\overset{N-1-\left|k\right|}{\underset{n=0}{\sum }}z\left(n\right)y_{c}\left(n-k\right)\right)}^{2}},$$

where:

(2) $$y_{s}\left(n\right)=x\left(n\right)\sin\left(2\pi f_{c}n\right),\;y_{c}\left(n\right)=x\left(n\right)\cos\left(2\pi f_{c}n\right),$$

where: x(n)—received signal, z(n)—represents a pseudo-random sequence, $f_{c}$—carrier frequency, n—discreet time (sample).

Next, a decision is made as to whether or not there is a preamble, based on the maximum value of the correlation and the mean values in its surroundings. If this process fails, the recorded fragment is discarded, the next fragment is registered, and the preamble detection procedure is repeated. After detecting the preamble, the reception is carried out. Based on the information about the preamble time position in the recorded signal, the signal is split into time slices according to the configuration data. Each of the time fragments is transformed into the frequency domain. Prior to performing the Fourier transform, the time signal is zero-padded to obtain the desired frequency resolution at the receiver, and additionally it is windowed using a Hann’s window to minimize leakage. In the next step, diversity combining takes place. It consists of summing the spectra corresponding to the individual symbols obtained from the receiving paths. The summing is performed with the same weights after normalizing the spectra to the value 1 in the band in which the data transmission takes place. This is to prevent one of the receivers from unduly influencing the final result, which may be caused by, e.g., higher receiver sensitivity or higher gain in the receiving path. The use of the diversity combining technique is primarily to counteract the occurrence of selective fading. The fading, caused by multipath propagation, may lead to a situation in which it will not be possible to receive data on a given frequency. This would cause an increase in the bit error rate (BER). Depending on the phase difference of the incoming signals, the met signal may be amplified, completely suppressed or an intermediate situation may occur. When the path distance difference Δr is equal to an odd multiple of the wavelength λ, there is complete suppression, which can be written as [20]:

(3) $$\frac{\lambda }{2}\left(2n+1\right)=\Delta r,\;n\in N,$$

Knowing that:

(4) $$\lambda =\frac{c}{f},$$

where: c—speed of the wave in propagation environment, f—frequency of the wave, can be written as:

(5) $$f=\frac{c\left(2n+1\right)}{2\Delta r}.$$

The frequency at which the fading occurs repeats with a period equal to c/Δr.

As described in [21], the second advantage of using diversity combining in the presented method, consisting of summing the spectra from individual reception paths, is the reduction of the variance of the components of the resultant amplitude spectrum derived from noise. After the diversity combining, the spectra obtained for individual symbols are decoded. The decoding consists of making a decision about the value of the transmitted bit on the basis of examining the dependence of the amplitude values between the spectrum components on which the bit is emitted, to investigate whether, for a given pair of frequencies at which a single bit is transmitted, which frequency component (lower or higher) has the greater amplitude. Depending on that, the value of 0 or 1 is assumed as the transmitted data. No single components are examined, but those components coming from the surrounding frequency at which transmission of a given bit are expected.

## 3. Model of the Multipath Channel

Let the discrete hydroacoustic signal x(n) and the channel impulse response (CIR) h(n) of the multipath channel in the SISO (single input single output) system be given. Assuming that the channel is stationary in the considered time interval, the received signal can be expressed as a sum of M multipath components of a certain amplitude $a_{i}$ reaching the receiver with a certain delay $n_{i}$ in the presence of noise w(n), i.e., in the form:

(6) $$y\left(n\right)=\sum_{i=1}^{M}a_{i}x\left(n-n_{i}\right)+w\left(n\right),$$

where: n—a discrete time (sample).

The impulse response characterizes the propagation conditions of the acoustic wave along the path between the transmitter and receiver, including multipath conditions. The form of this response will depend on the mutual spatial position of the transmitter and receiver in the water area. Accordingly, the received signal may be represented as a convolution of the transmitted signal, with the impulse response of the channel in the presence of noise w(n) [22]:

(7) y(n)=h(n)∗x(n)+w(n).

In the case of a SIMO (single input multiply output) system, i.e., with one transmitter and K receivers, the relationship between the output and input signals can be expressed as a matrix equation [20]:

(8) y(n)=(H∗x)(n)+w(n),

where: vector y(n) having a dimension of (K×1) contains instantaneous values of signals received at time n by individual hydrophones, vector w(n) having a dimension of (K×1) represents instantaneous noise values at time n in each receiving channel, vector H(n) having a dimension of (K×1) describes the propagation conditions of the hydroacoustic channel, where the elements $h_{1}\left(n\right),\ldots ,h_{K}\left(n\right)$ of this vector represent the impulse response of the hydroacoustic channel between the transmitting hydrophone and the k-th receiving hydrophone.

In wireless communication systems distributions such as Rayleigh, Weibull, Nakagami are commonly used to model propagation conditions.

In the matrix model given by (8), a vector w(n) was introduced, representing the noise recorded in each receiver. Most often it is assumed that the noise has a Gaussian distribution, where the real part and the imaginary part of the noise w(n) are independent random variables with zero mean value and variance $\frac{\sigma_{w}^{2}}{2}$ [20]:

(9) $$\{\begin{array}{c} Re\left[w\left(n\right)\right]∼N\left(0,\frac{\sigma_{w}^{2}}{2}\right)\\ Im\left[w\left(n\right)\right]∼N\left(0,\frac{\sigma_{w}^{2}}{2}\right) \end{array},$$

The power of such defined noise in each receiver will be the same, i.e., $\sigma_{w}^{2}$.

The above analysis concerned the time domain. In the proposed method of diversity combining, the signal spectra from individual hydrophones are summed up. Then the variance $\sigma_{f}^{2}$ of the noise observed in the spectrum of the diversity combined signal decreases according to the equation [21]:

(10) $$\sigma_{f}^{2}=\frac{2^{1/\left(2L\right)}\;{\left(\sigma_{w}^{2}\right)}^{2}}{M},$$

where: M—the number of spectrum components, L—the number of spectra used to derive the mean value of the spectrum.

## 4. Description of the Laboratory Stand

**Figure 4:**
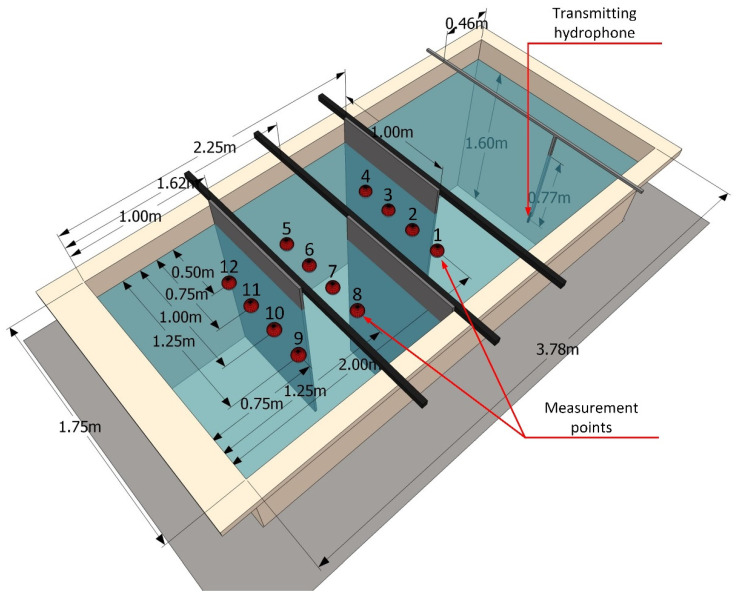
Illustrative drawing of the water tank and measuring conditions.

**Figure 5:**
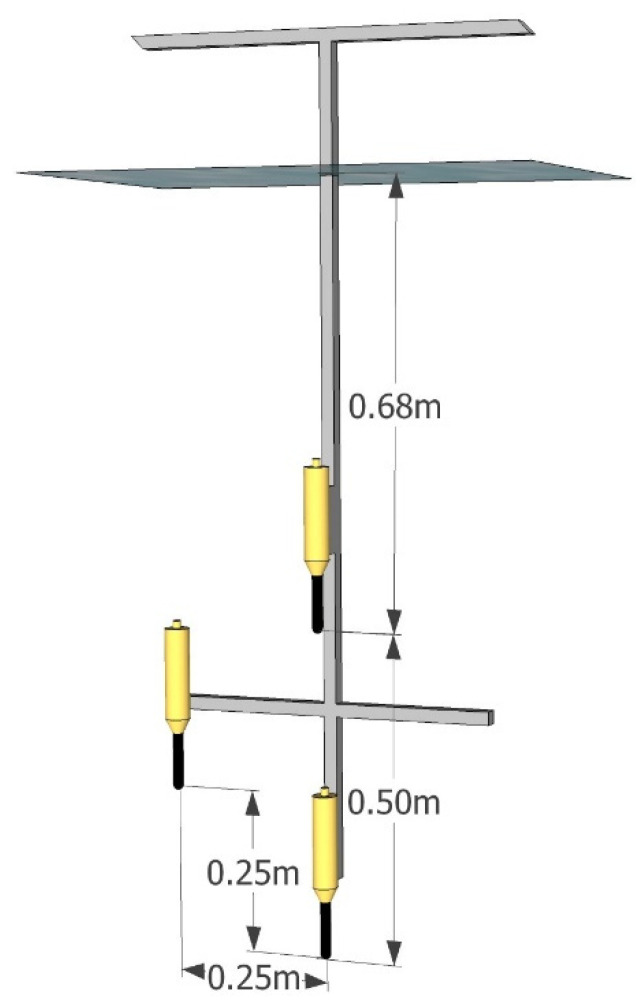
Illustrative drawing of the set of receiving hydrophones.

The tests were carried out in a laboratory tank shown in Figure 4. The walls of the tank were smooth, made of polypropylene (PP) with a thickness of 10 mm. The internal dimensions of the tank were: 3.78 m × 1.75 m × 1.8 m. The water column was 1.6 m high. The tank was divided into four compartments to achieve NLOS conditions. Acoustic panels AKU-PRO 140 made of material with a density of 140 kg/m³ and a thickness of 20 mm were used as partitions. The edge of the panels adjoined one of the tank walls and the bottom. The upper edge protruded above the water level. In this way, an artificial corridor was created, reflecting, for example, the interior of the wreck. On one side of the tank, in front of the compartments, a Reson TC4013 transmitting hydrophone was placed. The signal to this hydrophone was fed from a PC via a digital-to-analog NI USB6366 card and an Etec PA1001 power amplifier. Three Reson TC4014 hydrophones were placed on the receiving path. Their spatial configuration is shown in Figure 5. The hydrophones were powered by 12 V, and the signals were fed through the NI USB6366 analog-to-digital converter card to a PC, where they were recorded and processed. The software of both the transmitter and the receiver was implemented in the Matlab environment.

The measurements were performed by locating the receiving hydrophones at 12 selected points, which are marked in Figure 4. At each point, 10 transmissions were made. In each transmission 10,000 bits were sent.

## 5. Results

### 5.1. Propagation Conditions

**Figure 6:**
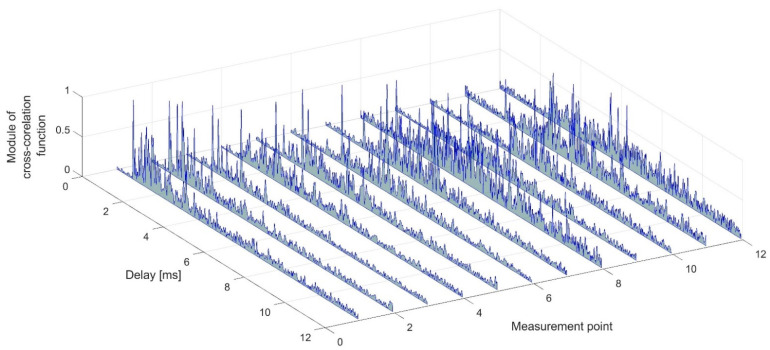
Modules of estimates of impulse responses at measurement points.

In order to determine the propagation conditions for individual measurement points, using the transmitted preamble, the impulse responses were estimated using the correlation method as described in [23]. The module form of the impulse response estimates at individual measurement points is shown in Figure 6. The presented estimates were normalized with respect to the maximum value, separately for each measurement point. It is clearly visible that, as the set of receiving hydrophones is moved in the created corridor, LOS conditions are present at the first measuring point, and NLOS conditions prevail in the subsequent points. It can be determined on the basis of the appearance time of the peak of the estimation of impulse response, i.e., only in the first measurement point the signal arriving directly has the highest power.

**Figure 7:**
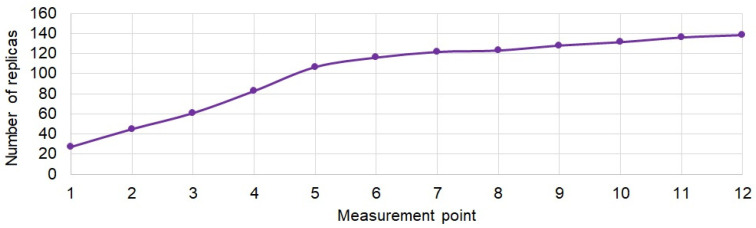
Average number of replicas at measurement points.

Based on the determined estimates of the channel impulse responses, in accordance with the recommendations of ITU-R P.1407-7 [24], the average number of replicas at each measurement point was determined. The results are shown in Figure 7. The results show that as the receiver set is moved deeper into the corridor, the average number of replicas increases significantly, even above 100 at the last measurement point. This clearly demonstrates the conditions of strong multipath signal propagation.

Using the power profile of the received signals as a function of delay τ (PDP—power delay profile), it is possible to determine the total power $P_{m}$ of all signal replicas registered at individual measurement points [24]:

(11) $$P_{m}=\sum_{i=1}^{N}p\left(\tau_{i}\right),$$

where $p\left(\tau_{i}\right)$ is power of i-th replica, N—amount of replicas.

This allows the average delay of received replicas to be calculated according to the dependency [24]:

(12) $$\bar{\tau }=\frac{\sum_{i=1}^{N}\tau_{i}p\left(\tau_{i}\right)}{P_{m}}.$$

On this basis, the root mean square delay spread was determined according to the equation [24]:

(13) $$\tau_{rms}=\sqrt{\frac{\sum_{i=1}^{N}\tau_{i}^{2}p\left(\tau_{i}\right)}{P_{m}}-{\bar{\tau }}^{2}}.$$

**Figure 8:**
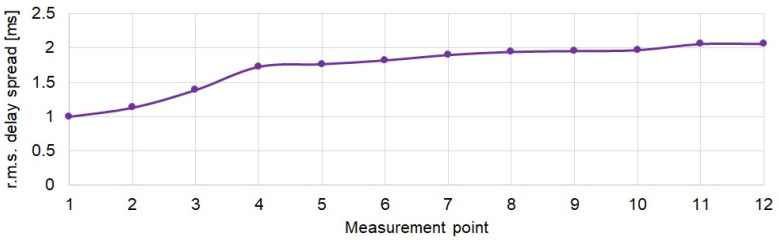
Root mean square delay spread $\tau_{rms}$ at measurement points.

The obtained results are shown in Figure 8.

The $\tau_{rms}$ delay spread increases significantly from approx. 1 ms at the first measurement points to almost 1.8 ms at 4th measurement point. In the following measurement points, this increase is much smaller.

**Figure 9:**
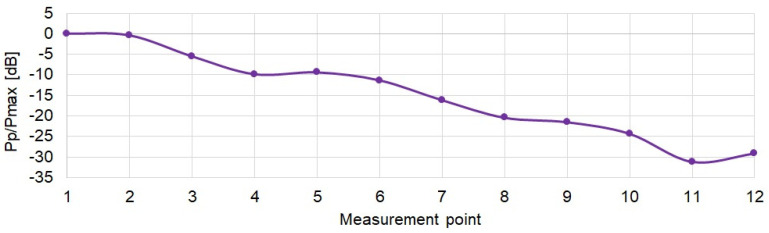
Change of signal power at measurement points.

The power relations between the recorded signals at individual measuring points were determined. Changes in the power of received signals at individual measurement points are shown in Figure 9, where $P_{max}$ is the maximal measured power from all measurement points and $P_{p}$ is the measured power in p-th point. As expected, the signal power decreases by up to 30 dB as moving down the corridor.

**Figure 10:**
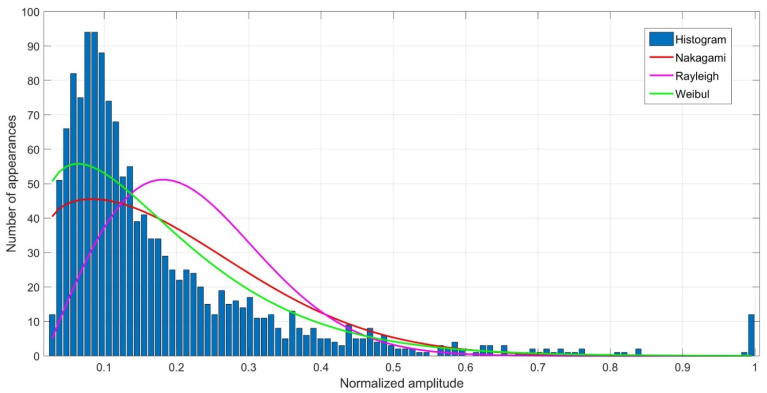
Histogram of normalized amplitudes of replicas. Probability density functions of replica amplitudes for Weibull, Rayleigh and Nakagami distributions at measurement point No. 5.

The distributions of replicas amplitudes at individual measurement points were determined. For this purpose, normalized amplitude histograms were created and compared with the Weibull, Rayleigh and Nakagami distributions. An example of the comparison of the obtained histogram from 12 impulse response measurements with the adopted distributions for the measurement point No. 5 is shown in Figure 10.

In order to assess the adequacy of the considered distributions to describe the statistical properties of the replica amplitudes, the so-called non-dimensional error index (NDEI) defined as the ratio of the root mean squared error to the standard deviation of the histogram:

(14) $$\mathrm{NDEI}=\frac{\sqrt{\frac{1}{N}\sum_{i=1}^{N}{\left(h_{i}-\hat{p}\right)}^{2}}}{\sqrt{\frac{1}{N-1}\sum_{i=1}^{N}{\left(h_{i}-\bar{h}\right)}^{2}}},$$

where: $h_{i}$—the histogram value of the normalized replica amplitudes for i-th measurement, $\hat{p}$—probability density estimator, $\bar{h}$—mean value of the histogram, N—number of intervals in the histogram. For each measurement points, the assessment was made and the NDEI error was determined for each individual distribution, as shown in Table 1.

The results summarized in the Table 1 show that at each measurement point, the lowest value of the NDEI error was obtained for the Weibull distribution, making it the distribution that best reflects the propagation conditions in the laboratory tests. This conclusion is similar to that presented in [13].

### 5.2. The Transmission Quality in Laboratory Conditions

When assessing the transmission quality, the bit error rate for reception from single hydrophones and the diversity combining from two and three reception paths was determined. The tests were carried out with the following transmission parameters: $N_{sf}=5$, $N_{bs}=2000$, $F_{d}=10\;\mathrm{kHz}$, $\Delta f_{t}=20\;\mathrm{Hz}$, $\Delta f_{bits}=80\;\mathrm{Hz}$, $\Delta f_{r}=1\;\mathrm{Hz}$, $\tau_{guard}=0.04\;s$, $F_{c_{preamble}}=55\;\mathrm{kHz}$, $N_{preamble}=511$, $S_{preamble}=20\;\mathrm{kBd}$. The sampling frequency was 500 kHz (simultaneous). According to the above parameters, the symbol duration was 50 ms, the bandwidth occupied was 10–170 kHz, and the transmission rate was 19.397 kbit/s.

**Figure 11:**
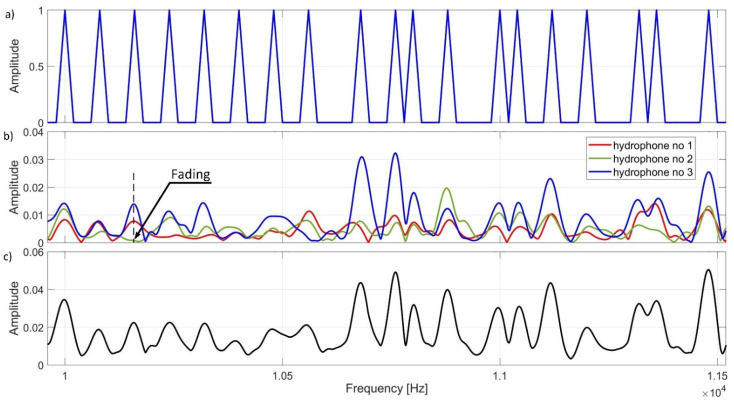
An example of fragments of amplitude spectrums (**a**) transmitted signal, (**b**) received signals, (**c**) after diversity combining.

Figure 11 shows an example of a fragment of the spectrum of the signal transmitted and received at measurement point number 3. Figure 11a shows the spectrum of transmitted signal for 19 bits. In Figure 11b, the spectrum of the signal received by individual hydrophones for the same 19 bits is shown. Figure 11c shows the spectrum after diversity combining. The obtained result indicates that, at the same frequencies, each of the hydrophones received a signal of a different strength. At some measurement points, we observe multipath-induced decays. In Figure 11b, the special case where the hydrophone No. 2 did not register the signal at a certain frequency is marked with an arrow. It should be noted that in the case of diversity combining, due to summing the spectra from the three reception paths, it is possible to detect all bits.

**Figure 12:**
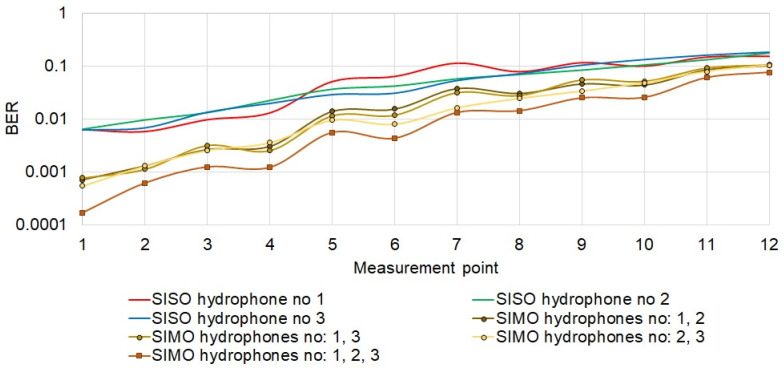
Quality of data reception.

Figure 12 shows the result of the bit error rate for the individual measurement points. At individual measurement points, the results obtained from individual hydrophones are similar to each other, i.e., a similar error rate was obtained. As the set of the receiving hydrophones was moved further down the corridor, the reception quality expressed in BER deteriorated. Diversity combining improves BER in all cases. As expected, the lower BER was obtained using three hydrophones. It should be noted that the greatest gain from diversity combining, even two orders of magnitude, was obtained at the measurement points located near the entrance to the corridor. For the points at the end of the corridor, the gain from diversity combining was two times smaller.

**Figure 13:**
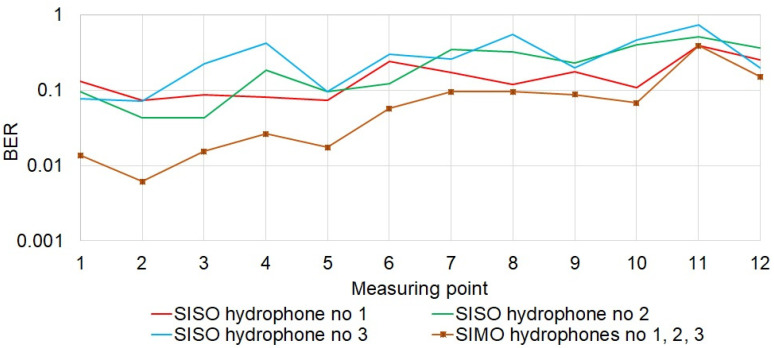
Quality of data reception for DSSS transmission.

For comparison, an experiment was carried out in which the DSSS transmission with BPSK modulation [22] was performed under the same propagation conditions as the MFSK transmission described earlier. The transmission parameters were as follows: sampling frequency 500 kHz, carrier frequency 55 kHz, modulation rate 20 kHz, 4 chips per bit, therefore the transmission speed was about 5 kbit/s. This speed is comparable to the speed of the signal transmission in the method used in the present work, assuming the same bandwidth. Assuming that the bandwidth occupied by the MFSK signal would be 40 kHz then it would give a transmission rate of 4.85 kbit/s, which is comparable to the speed of the performed DSSS transmission. The results of the experiment are shown in Figure 13.

Figure 13 shows that the diversity combining carried out in accordance with the method for DSSS signals described in [22] resulted in the improvement of the reception quality. However, comparing the obtained BER error rates with the results presented in Figure 12, the proposed method based on MFSK is much better than the traditional DSSS. This is mainly due to the greater resistance of the MFSK method to the effects of the multipath phenomenon.

### 5.3. Simulation Research

**Figure 14:**
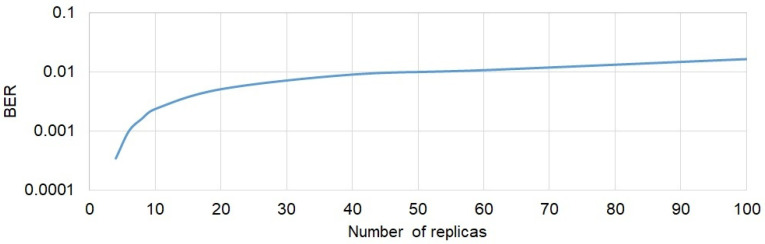
The impact of the number of replicas on the bit error rate.

The error in making a decision about the transmitted bit in the individual receive path can be caused by selective fading and/or noise. Selective fading is caused only by multipath propagation. In order to assess the impact of this phenomenon on the reception quality expressed in BER, a simulation using the Monte Carlo method and MFSK modulation was carried out. The mathematical model presented in Section 3 was used in this simulation. The influence of the number of replicas on the probability of a wrong decision was checked. In the simulation, the number of replicas was changed in the range of 2 to 100. In all cases, the total power of the replicas was the same and the amplitudes of the replicas were adopted according to the Weibull distribution. In Weibull distribution, the probability of occurrence of an amplitude of value 1 (maximum value) is very low. However, in received signals (Figure 10) there were always replicas with such amplitude. In order to reflect the real conditions in the simulated impulse response of the channel, before normalization, a signal with the maximum value equal to 1 was introduced so as to obtain the result as shown in the histogram presented in Figure 10. The simulation was performed without the presence of noise. The results of the above simulation are presented in Figure 14.

A significant deterioration in the quality of reception is observed when increasing the number of replicas to a value of about 20. A further increase in the replica number does not cause a significant increase in BER. Taking into account the average number of replicas at individual measurement points (Figure 7), it can be concluded that the reception quality obtained by the simulation (Figure 14) is consistent with that measured in the laboratory conditions presented in Figure 12. For example, the average number of replicas at point No. 5 was about 100. At this point, the measured BER was about 0.02, which is a good agreement with the simulation result (Figure 14).

## 6. Conclusions

Wireless data transmission in water environment is a non-trivial problem. Additionally, complications arise when NLOS conditions occur. Considering the demand for communication systems operating in such conditions, the possibilities of wireless underwater communication in the absence of direct visibility of the hydrophones were tested in this work. The tests were carried out in laboratory conditions simulating the situation which, for example, could be met in a wreck. By examining the impulse responses, it was found that NLOS conditions were indeed achieved in the experimental tank. The determined spectrum of received signals confirmed the presence of frequency-selective fading in the prepared artificial corridor in the laboratory tank. In the adopted measurement scenario, it was shown that the number of replicas increases with movement down the corridor, while the root mean square delay spread does not increase significantly. We found that the proposed technique of signal formation and diversity combining presented in this paper ensured a high quality of transmission compared to the DSSS technique. As expected, diversity combining greatly improves the transmission quality.

The original qualities of this paper include: the impulse response studies in NLOS conditions, the evaluation of the quality of diversity combining in conditions of strong multipath propagation using the MFSK technique of forming the transmitted signal.

The presented research results provide the basis for undertaking work leading to a communication system with autonomous underwater vehicles which operate in difficult propagation conditions, such as wrecks and ports.

## Figures and Tables

**Table 1:** NDEI error for Weibull, Reyleigh, Nakagami distributions at individual points.

Measurement Point	Weibull	Rayleigh	Nakagami
1	0.517	0.93266	0.66894
2	0.77901	0.96381	1.0891
3	0.87935	1.081	1.2216
4	0.89289	1.0866	1.1133
5	0.42217	0.80168	0.53029
6	0.44172	0.83668	0.58009
7	0.46689	0.76069	0.54274
8	0.4166	0.77924	0.53395
9	0.38014	0.5733	0.44018
10	0.48478	0.63987	0.53011
11	0.45607	0.75739	0.56195
12	0.58868	0.82631	0.66981

## Data Availability

Not applicable.

## References

[1] Piskur P., Szymak P., Jaskólski K., Flis L., Gąsiorowski M. (2020). Hydroacoustic System in a Biomimetic Underwater Vehicle to Avoid Collision with Vessels with Low-Speed Propellers in a Controlled Environment. Sensors.

[2] Kochanska I., Schmidt J.H., Schmidt A.M. (2021). Study of probe signal bandwidth influence on estimation of coherence bandwidth for underwater acoustic communication channel. Appl. Acoust..

[3] Czapiewska A., Luksza A., Studanski R., Zak A. (2020). Reduction of the Multipath Propagation Effect in a Hydroacoustic Channel Using Filtration in Cepstrum. Sensors.

[4] Pavan Ganesh P.S.S., Venkataraman H. (2020). E-CRUISE: Energy-based throughput analysis for cluster-based RF shallow underwater communication. IET Commun..

[5] Chen Y., Shen W., Li Z., Hu C., Yan Z., Jiao Z., Gao J., Cao M., Sun K., Jin X. (2020). Underwater transmission of high-dimensional twisted photons over 55 meters. PhotoniX.

[6] Zhu S., Chen X., Liu X., Zhang G., Tian P. (2020). Recent progress in and prespectives of underwater wireless optical communication. Prog. Quantum Electron..

[7] Sun X., Kong M., Alkhazragi O., Shen C., Ooi E., Zhang X., Buttner U., Ng T.K., Ooi B.S. (2020). Non-line-of-sight methodology for high-speed wireless optical communication in highly turbid water. Opt. Commun..

[8] Stojanovic M., Catipovic J., Proakis J. (1993). Adaptive multichannel combining and equalization for underwater acoustic communications. J. Acoust. Soc. Am..

[9] van Walree P.A., Socheleau F.X., Otnes R., Jenserud T. (2017). The Watermark Benchmark for Underwater Acoustic Modulation Schemes. IEEE J. Ocean. Eng..

[10] Çavuslu M.A., Altuncu M.A., Özcan H., Gülagiz F.K., Sahin S. (2021). Estimation of underwater acoustic channel parameters for Erdek/Turkey region. Appl. Acoust..

[11] Liu B., Jia N., Huang J., Guo S., Xiao D., Ma L. (2022). Autoregressive model of an underwater acoustic channel in the frequency domain. Appl. Acoust..

[12] Li G., Wu J., Tang T., Chen Z., Chen J., Liu H. (2019). Underwater Acoustic Time Delay Estimation Based on Envelope Differences of Correlation Functions. Sensors.

[13] Kulhandjian H., Melodia T. Modeling underwater acoustic channels in short-range shallow water environments. Proceedings of the 9th ACM International Conference on Underwater Networks and Systems, WUWNET 2014.

[14] Yuan F., Jia Z., Cheng E. (2020). Chirp-rate quasi-orthogonality based DSSS-CDMA system for underwater acoustic channel. Appl. Acoust..

[15] Pranitha B., Anjaneyulu L. (2020). Analysis of Underwater Acoustic Communication System Using Equalization Technique for ISI Reduction. Procedia Comput. Sci..

[16] Kaczorek P., Studanski R., Zak A. (2017). Data transmission in the hydroacoustic channel–experimental researches. J. Mar. Eng. Technol..

[17] Kochanska I., Salamon R., Schmidt J.H., Schmidt A.M. (2021). Study of the Performance of DSSS UAC System Depending on the System Bandwidth and the Spreading Sequence. Sensors.

[18] Fang T., Liu S., Ma L., Zhang L., Khan I.U. (2021). Subcarrier modulation identification of underwater acoustic OFDM based on block expectation maximization and likelihood. Appl. Acoust..

[19] Kochanska I. (2020). Reliable OFDM Data Transmission with Pilot Tones and Error-Correction Coding in Shallow Underwater Acoustic Channel. Appl. Sci..

[20] Kozłowski S. (2010). Analysis and Investigation of MIMO Systems Utilizing Adaptive Array Antennas. Ph.D. Thesis.

[21] Studanski R., Wąs R. (2007). Detection of DS CDMA signals by power density spectrum analysis. Sci. J. Fac. Electron. Telecommun. Inform. Gdan. Univ. Technol..

[22] Czapiewska A., Luksza A., Studanski R., Zak A. (2020). Application of Diversity Combining with RLS Adaptive Filtering in Data Transmission in a Hydroacoustic Channel. Sensors.

[23] Studanski R., Zak A. (2017). Results of impulse response measurements in real conditions. J. Mar. Eng. Technol..

[24] Recommendation ITU-R P (2019). 1407-7 Multipath Propagation and Parameterization of Its Characteristics. https://www.itu.int/rec/R-REC-P.1407-7-201908-I/en.

